# GE11 peptide conjugated selenium nanoparticles for EGFR targeted oridonin delivery to achieve enhanced anticancer efficacy by inhibiting EGFR-mediated PI3K/AKT and Ras/Raf/MEK/ERK pathways

**DOI:** 10.1080/10717544.2017.1386729

**Published:** 2017-10-11

**Authors:** Jiang Pi, Jinhuan Jiang, Huaihong Cai, Fen Yang, Hua Jin, Peihui Yang, Jiye Cai, Zheng W. Chen

**Affiliations:** a State Key Laboratory of Quality Research in Chinese Medicines, Macau University of Science and Technology, Macau, PR China;; b Department of Microbiology and Immunology, University of Illinois at Chicago, Chicago, IL, USA;; c Department of Chemistry, Jinan University, Guangzhou, PR China

**Keywords:** Selenium nanoparticles, GE11 peptide, oridonin, delivery, EGFR targeting

## Abstract

Selenium nanoparticles (Se NPs) have attracted increasing interest in recent decades because of their anticancer, immunoregulation, and drug carrier functions. In this study, GE11 peptide-conjugated Se NPs (GE11-Se NPs), a nanosystem targeting EGFR over-expressed cancer cells, were synthesized for oridonin delivery to achieve enhanced anticancer efficacy. Oridonin loaded and GE11 peptide conjugated Se NPs (GE11-Ori-Se NPs) were found to show enhanced cellular uptake in cancer cells, which resulted in enhanced cancer inhibition against cancer cells and reduced toxicity against normal cells. After accumulation into the lysosomes of cancer cells and increase of oridonin release under acid condition, GE11-Ori-Se NPs were further transported into cytoplasm after the damage of lysosomal membrane integrity. GE11-Ori-Se NPs were found to induce cancer cell apoptosis by inducting reactive oxygen species (ROS) production, activating mitochondria-dependent pathway, inhibiting EGFR-mediated PI3K/AKT and inhibiting Ras/Raf/MEK/ERK pathways. GE11-Se NPs were also found to show active targeting effects against the tumor tissue in esophageal cancer bearing mice. And in nude mice xenograft model, GE11-Ori-Se NPs significantly inhibited the tumor growth via inhibition of tumor angiogenesis by reducing the angiogenesis-marker CD31 and activation of the immune system by enhancing IL-2 and TNF-α production. The selenium contents in mice were found to accumulate into liver, tumor, and kidney, but showed no significant toxicity against liver and kidney. This cancer-targeted design of Se NPs provides a new strategy for synergistic treating of cancer with higher efficacy and reduced side effects, introducing GE11-Ori-Se NPs as a candidate for further evaluation as a chemotherapeutic agent for EGFR over-expressed esophageal cancers.

## Introduction

Up to now, cancer is still one of the leading cause of death worldwide, accounting for millions of death annually, is a major threat to the health of human beings, which urges more and more attentions to be paid for cancer treatment. As one of the most widely operated methods for cancer treatment currently, chemotherapy based new drug system design, especially the targeted drug delivery systems, has become one of the main trend for cancer therapy because the conventional anti-cancer agents used in chemotherapy always cannot demonstrate predominant tumor specificity compared to normal cells. Nanotechnology-based drug delivery with specific surface modification of nanomaterials for cancer cell targeting, has emerged to provide more efficient delivery of drugs to tumors by ligand-receptor recognition and passive accumulation, which is known as the enhanced permeability and retention (EPR) effect (Sun et al., 2014b).

Taking the advantages of the native anticancer effects (Kong et al., 2011; Pi et al., 2013), drug carrier role (Liu et al., 2012; Li et al., 2016), protection effects (Wang et al., 2005; Li et al., 2011b), anti-angiogenesis effects (Sun et al., 2013; Sun et al., 2014a), and anticancer activity enhancement effects (Huang et al., 2013; Gao et al., 2014) for chemotherapy drugs, selenium nanoparticles (Se NPs) have been highlighted as a kind of potential candidates for the design of nanoparticle-based drug delivery system. By conjugation with cancer cell targeting biomolecules, such as transferrin (Huang et al., 2013), RGD peptide (Fu et al., 2016), folate (Liu et al., 2015a), and gracilaria lemaneiformis polysaccharide (Jiang et al., 2014), Se NPs could selectively deliver anticancer drugs into cancer cells to achieve enhanced anticancer activity. Generally, surface modification of Se NPs by receptor-specific ligands or antibodies can bind to the receptors on cancer cell surface and undergo cellular internalization via a receptor-mediated endosomal–lysosomal process; subsequently, the nanoparticle can undergo degradation or destabilization in cells, leading to intracellular release of the drugs to achieve anticancer synergism combing with the native anticancer activity of Se NPs (Liu et al., 2012; Huang et al., 2013).

Epidermal growth factor receptor (EGFR), a tyrosine kinases receptor (RTK) belongs to the ErbB family, has been found to be over-expressed in many types of cancer, making it a typical target for cancer therapy (Salomon et al., 1995; Mendelsohn & Baselga, 2000). EGFR has also been reported to be over-expressed in esophageal squamous cell carcinoma (ESCC), a kind of cancer arising from the esophagus – the food pipe that runs between the throat and the stomach (Wang et al., 2014; Cui et al., 2015). Thus, anti-EGFR targeted therapy may have certain curative effect or generate breakthrough for esophageal cancer therapy (Zhao & Chen, 2014). The synthetic 12 amino acids peptide GE11, with the sequence of Y-H-W-Y-G-Y-T-P-Q-N-V-I, has been proved to be an efficient EGFR targeting peptide *in vitro* and *in vivo* (Li et al., 2005; Master et al., 2012; Mickler et al., 2012; Xu et al., 2013), introducing it as one of the best candidates for the design of EGFR-targeted drug delivery system.

Oridonin, an ent-kaurane diterpenoid isolated from Rabdosia rubescens, is a kind of traditional Chinese medicine widely used for pharyngitis and esophageal cancer treatment for hundreds of years. As the main pharmacological active substance of Rabdosia rubescens, oridonin has been proved to obtain strong anticancer activity against different types of cancer cells (Li et al., 2011a; Zhao & Chen, 2014), also including esophageal cancer cells (Liu & Yue, 2014; Pi et al., 2017). On the strength of its anticancer activity, the oridonin injection has been introduced for clinical cancer treatment in China, but the high toxicity against normal cells induced by weak targeting effects of oridonin for cancer cells and the poor water solubility still restrict its clinical use (Chen et al., 2011). Due to the strong native anticancer activity of Rabdosia rubescens and oridonin against esophageal cancer, and taking the advantages of the drug delivery and anticancer activity of Se NPs, we aimed to develop a kind of more effective anticancer system loading with oridonin for esophageal cancer treatment. Therefore, esophageal cancer cell line was used as the cancer cell model for the following study.

To this end, we developed Se NPs conjugated with EGFR-binding peptide GE11 to encapsulate and deliver oridonin for esophageal cancer treatment. The oridonin-loaded GE11 conjugated Se NPs (GE11-Ori-Se NPs) could enhance the cytotoxicity of oridonin against cancer cells to show stronger anticancer efficiency with decreased toxicity against normal cells. The *in vitro* cellular uptake mechanism and anticancer activity of GE11-Ori-Se NPs, and the underlying molecular mechanisms were also investigated in this study. *In vivo* investigation of nanoparticles on nude mice bearing esophageal cancer xenografts further indicated that GE11-Ori-Se NPs possessed high antitumor efficiency with no systemic toxicity, throwing light on the use of GE11-Ori-Se NPs to be a viable drug candidate for esophageal cancer treatment.

## Materials and methods

### Materials

Sodium selenite, oridonin, EDC, ascorbic acid, chitosan, dialysis bags (MWCO: 8000–14,000), nitric acid, and hydrogen peroxide were obtained from Ming Wang Biotechnology (Dalian, China). GE11 polypeptide was purchased from GL Biochem Ltd. (Shanghai, China). Lyso Tracker red kits, paraformaldehyde, coumarin-6, sodium azide (NaN_3_), 2-deoxy-D-glucose (DOG), sucrose, dynasore, and nystatin were obtained from Sigma-Aldrich (St. Louis, MO). FBS, penicillin/streptomycin, DMEM medium, and trypsin kit were purchased from Gibco Laboratories (Gaithersburg, MD). The Bio-Rad protein assay kit was obtained from Bio-Rad (Hercules, CA) and the HRP chemiluminescent substrate reagent kit was obtained from Invitrogen (Waltham, MA). RIPA lysis buffer, Bcl-2 antibody, Bax antibody, EGFR antibody, p-EGFR antibody, Ras antibody, Raf antibody, MEK antibody, p-MEK antibody, ERK antibody, p-ERK antibody, PI3K antibody, p-PI3K antibody, AKT antibody, p-AKT antibody, β-actin antibody, anti-mouse IgG and anti-rabbit IgG were from Cell Signaling Technology (Danvers, MA). Caspase-3 and Caspase-9 detection kits, 3-(4,5)-dimethylthiazo(-z-y1)-3,5-di-phenytetra-zoliumromide (MTT), Annexin V-FITC/PI apoptosis detection kit, reactive oxygen species (ROS) detection kit, DAPI, and cell cycle analysis kits were purchased from Beyotime Institute of Biotechnology (Shanghai, China). Mice TNF-α and IL-2 ELISA kits were purchased from Huamei (Wuhang, China). Matrigel was obtained from BD (East Rutherford, NJ). All reagents used in the experiments were of analytical grade.

### Preparation and characterization of GE11-Ori-Se NPs

An aliquot of 200 μL sodium selenite (50 mM) solution and 600 μL chitosan solution (0.5%) were mixed with 100 μL oridonin solution (50 mM) to form mixture. Then, 1600 μL ascorbic acid solution (50 mM) was added to the mixture and Milli-Q water was added to obtain a final volume of 10 mL for overnight reaction at 4 °C. The excess reactants were removed by dialysis against Milli-Q water until no Se was detected in the outer solutions as determined by inductively coupled plasma atomic emission spectrometry (ICP-AES) analysis to obtain oridonin loaded Se NPs (Ori-Se NPs). Chi-Se NPs were prepared using the similar method as described for Ori-Se NPs preparation except that no oridonin was added into the mixture. To obtain GE11-Ori-Se NPs, Ori-Se NPs was further mixed with 40 μL EDC solution (500 mM) and 80 μL GE11 peptide solution (50 mg/mL) for overnight reaction at 4 °C. The excess reactants were removed by dialysis against Milli-Q water to obtain GE11-Ori-Se NPs. To obtain coumarin-6-loaded GE11-Se NPs, all preparation processes were similar except that 100 µL coumarin-6 solution was also added into the mixture of sodium selenite and chitosan, and then surface modified with GE11 peptide. Coumarin-6-loaded Chi-Se NPs were prepared similar with coumarin-6-loaded GE11-Se NPs except that no further GE11 surface modification of the nanoparticles. The Se contents in different kinds of Se NPs were determined by ICP-AES analysis. The as-prepared GE11-Ori-Se NPs were characterized by several microscopic and spectroscopic measurements including transmission electron microscope (TEM), Fourier transform infrared spectroscopy (FTIR), UV–vis spectroscopy. The obtained GE11-Ori-Se NPs solution was dropped onto the copper net and dried at room temperature for 12 h. TEM images and TEM-EDS data were taken by using a TEM (Philips, Amsterdam, Holland). To determine the chemical composition of nanoparticles, FTIR (Equinox 55 IR spectrometer) and UV–vis (Carry 5000 spectrophotometer) studies were also conducted, respectively. The size distribution and zeta potential of the nanoparticles were determined by Dynamic Light Scattering (DLS, Malvern Instruments, Malvern, United Kingdom).

### *In vitro* drug release of GE11-Ori-Se NPs

An aliquot of 10 mg of the GE11-Ori-Se NPs (freeze dried) was suspended in 10 mL of PBS solution at different pH (7.4 and 5.5) with constant shaking at 37 °C in a hard glass tube. At a specific time following incubation, a specific amount of solution was taken out from the vial with pipet and the same volume of fresh PBS solution was added. The released oridonin was measured by high performance liquid chromatography (HPLC, Agilent, Santa Clara, CA), and the experiment was performed in triplicate. Then, 20 μL of sample solution was analyzed by HPLC equipped with a Luna C18 column (250 mm ×4.6 mm, 5 μm), the mobile phase was a mixture of water and methanol in the volume ratio of 46:54 with a flow rate of 1.0 mL/min, and the wavelength was set at 242 nm.

### Cell culture

Human esophageal cancer KYSE-150 cell line and EC9706 cell line are obtained from tumor cell library of Chinese Academy of Medical Sciences (Beijing, China). KYSE-150 and EC9076 cells are cultured with DMEM medium supplemented with 10% FBS, 100 U/mL penicillin, and 100 g/mL streptomycin in a humidified atmosphere of 5% CO_2_ at 37 °C. Human fibroblast-like synovial cells (FLS), isolated from the arthritis patients, were provided by Dr. Xiaohui Su (State Key Laboratory of Quality Research in Chinese Medicines, Macau University of Science and Technology, Macau, China) and cultured with DMEM medium supplemented with 20% FBS, 100 U/mL penicillin, and 100 g/mL streptomycin in a humidified atmosphere of 5% CO_2_ at 37 °C. THP-1 cells were purchased from ATCC (Manassas, VA) and cultured with 1640 medium supplemented with 10% FBS, 100 U/mL penicillin, and 100 g/mL streptomycin in a humidified atmosphere of 5% CO_2_ at 37 °C. To induce THP-1 cells into macrophages, cells were treated with phorbol-12-myristate-13-acetate (PMA, 100 nM) for 24 h.

### Cellular uptake of GE11-Se NPs

The cellular uptake of GE11-Se NPs in KYSE-150 cells, THP-1 cells, and FLS were analyzed by detecting the intracellular fluorescence of coumarin-6-loaded GE11-Se NPs using microplate reader. The cellular uptake of coumarin-6-loaded Chi-Se NPs was also analyzed using the same method in KYSE-150 cells. The cells were seeded into 96 well plates with a density of 1 × 10^4^ cells/well for 24 h and incubated with different concentration of coumarin-6-loaded nanoparticles for designed times. Cells were washed with PBS and lysed with 0.5% Triton X-100 in 0.2 M NaOH solution. Fluorescence microplate reader was used to measure the fluorescence intensity from the coumarin-6-loaded nanoparticles inside the wells with excitation and emission wavelengths set at 430 and 485 nm, respectively. Standard curves for the nanoparticles were constructed by suspending different concentrations of coumarin-6-loaded GE11-Se NPs and coumarin-6-loaded Chi-Se NPs in a similar way as cell sample preparation, which showed *R*^2 ^= 0.9912 and *R*^2^ = 0.9901 for coumarin-6-loaded GE11-Se NPs and coumarin-6-loaded Chi-Se NPs, respectively. The uptake of nanoparticles by cells was calculated from the standard curve and expressed as the amount of nanoparticles (μg) taken up per 10^8^ cells.

### GE11 competition assay and anti-EGFR antibody blocking assay

For free GE11 peptide competition assay, KYSE-150 cells were seeded into 96 well plates with a density of 1 × 10^4^ cells/well for 24 h. Excess concentrations of GE11 peptide were added to the wells and incubated at incubator for 1 h. Then cells were further incubated with 80 µM coumarin-6-loaded GE11-Se NPs for 2 h. For anti-EGFR antibody block test, KYSE-150 cells were seeded into 96 well plates with a density of 1 × 10^4^ cells/well for 24 h. Anti-EGFR antibody (1:200) was added into the wells and incubated at incubator for 1 h. Then cells were further incubated with 80 µM coumarin-6-loaded GE11-Se NPs for 0.5H, 1 and 2 h, respectively. Cells were washed with PBS and lysed with 0.5% Triton X-100 in 0.2 M NaOH solution. Fluorescence microplate reader was used to measure the fluorescence intensity from coumarin-6-loaded GE11-Se NPs inside the wells with excitation and emission wavelengths set at 430 and 485 nm, respectively. The cellular uptake efficacy was expressed as the percentage of the fluorescence of the testing wells over that of the positive control wells.

### Mechanisms of cellular uptake of GE11-Se NPs

The cellular uptake mechanism of GE11-Se NPs in KYSE-150 cells was determined under different endocytosis inhibited conditions. The cells were seeded into 96 well plates with a density of 1 × 10^4^ cells/well for 24 h and pretreated with different endocytosis inhibitors for 1 h, except that nystatin was pretreated for 30 min. Final concentration of specific endocytosis inhibitors were listed as follows: NaN_3_ 10 mM, DOG 50 mM, sucrose 0.45 M, dynasore 80 μM and nystatin 10 μg/mL. Then cells were further incubated with 80 μM coumarin-6-loaded GE11-Se NPs for 2 h. The control cells were incubated with 80 μM of coumarin-6-loaded GE11-Se NPs without the addition of inhibitors. For investigation of energy-dependent pathways, the cells were treated in complete medium at 4 °C for 4 h, followed by coumarin-6-loaded GE11-Se NPs treatment for 2 h. Cells were washed with PBS and lysed with 0.5% Triton X-100 in 0.2 M NaOH solution. Fluorescence microplate reader was used to measure the fluorescence intensity from coumarin-6-loaded GE11-Se NPs inside the wells with excitation and emission wavelengths set at 430 and 485 nm, respectively. The cellular uptake efficacy was expressed as the percentage of the fluorescence of the testing wells over that of the control wells.

### Intracellular localization of GE11-Se NPs

The intracellular localization of GE11-Se NPs in KYSE-150 cells was investigated by fluorescence microscopy with specific staining of lysosomes. The cells were seeded at a density of 5 × 10^4^ into confocal dishes for 24 h incubation; cells were incubated with Lyso-Tracker (Thermo Fisher Scientific, Waltham, MA) red for 1 h. After that, cells were incubated with 80 μM of coumarin-6-loaded GE11-Se NPs for various periods of time. After washed with PBS for three times, a confocal microscopy (Zeiss, Oberkochen, Germany) was used to image the localization of coumarin-6-loaded GE11-Se NPs in KYSE-150 cells.

### Lysosomal membrane integrity measurement

The integrity of the lysosomal membrane was assessed qualitatively by confocal microscopes and quantitatively by flow cytometer. Cells were seeded onto 35 mm petri-dishes for 24 h and then incubated in complete medium with 5 μg/mL AO and 10% FBS for 15 min. After treated with 80 μM GE11-Se NPs for 24 h and rinsed with PBS, the cells were observed under confocal microscopy. Identical cells seeded in 6 well plates were stained with AO as above, and were digested, collected, and measured by flow cytometry using the PerCP channel (excitation: 488 nm, emission: 690 nm).

### Cell viability

Human esophageal cancer KYSE-150 cells, EC-9706 cells, and FLS were seeded at a density of 5 × 10^3^ into 96 well plates for 24 h incubation. THP-1 cells were seeded at a density of 1 × 10^4^ into 96 well plates for 24 h incubation with PMA stimulation. And then, cells were incubated with different concentration of GE11-Ori-Se NPs, Chi-Se NPs or oridonin for 48 h. After GE11-Ori-Se NPs treatment, MTT reagents (10 μL, 5 mg/mL) were then added into each well for 4 h incubation. Then, the medium was removed, and the cells were suspended in 150 μL DMSO to incubate 4 h. A spectrophotometer (Tecan Group AG, Männedorf, Switzerland) was used to test absorbance at 570 nm.

### Cell cycle, apoptosis, and mitochondria membrane potential measurements

Annexin V-FITC/PI apoptosis detection kit and cell cycle detection kit were used to detect the apoptosis and cell cycle distribution of KYSE-150 cells following the manufacturer’s instructions. The cells were seeded at a density of 1 × 10^5^ into 6 well plates for 24 h incubation, and then incubated with different concentration of GE11-Ori-Se NPs for 48 h. For apoptosis detection, the harvested KYSE-150 cells were washed triple with PBS, suspended in Annexin V binding buffer, incubated with FITC-labeled Annexin V, and PI for 5 min at room temperature in dark, and then immediately analyzed by flow cytometer (BD, East Rutherford, NJ). For cell cycle detection, the harvested KYSE-150 cells were harvested, washed with PBS, and fixed with 70% ethanol overnight at 4 °C. The fixed cells were washed three times with PBS, treated with RNase A, stained with propidium iodide (PI) 50 µg/mL for 30 min at 37 °C, and analyzed by a flow cytometer (BD, East Rutherford, NJ).

Rhodamine 123-based flow cytometry was used to determine the alterations of mitochondria membrane potential of KYSE-150 cells before and after GE11-Ori-Se NPs treatment. The cells were seeded at a density of 1 × 10^5^ into 6 well plates for 24 h incubation, and then incubated with different concentration of GE11-Ori-Se NPs. After 24 h GE11-Ori-Se NPs treatment, the harvested and washed KYSE-150 cells were incubated with rhodamine 123 for 60 min in dark at 37 °C. Flow cytometer was used to detect the fluorescence signal of rhodamine 123 after the cells were collected and washed twice with PBS (BD, East Rutherford, NJ).

### Intracellular ROS measurement

KYSE-150 cells were harvested by centrifugation, washed with PBS twice, and then resuspended in PBS. After that, the cells were then stained with the ROS detection kit at 37 °C for 30 min following the manufacturer’s protocol. Then the cells were incubated with GE11-Ori-Se NPs, oridonin or Chi-Se NPs at 37 °C for different periods of time. The intracellular ROS level was examined by detecting fluorescence intensity of cells using fluorescence microplate reader (excitation and emission wavelength set as 488 and 525 nm).

### Western blot analysis

After 48 h GE11-Ori-Se NPs treatment, KYSE-150 cells were incubated with lysis buffer containing protease inhibitors to obtain total cellular proteins. The protein concentration in the lysate was measured using the Bio-Rad protein assay kit according to the manufacturer’s guidelines. Proteins were then denatured by boiling at 100 °C for 10 min in sample buffer. The samples were then separated by electrophoresis on 10% SDS-polyvinylamide minigels, after which they were transferred to polyvinylidene difluoride membranes. The transferred membranes were blocked with 5% skim milk in TBST solution for 1 h, and then washed three times with TBST (5 min each time). After incubation with primary antibodies (Bcl-2 antibody, Bax antibody, EGFR antibody, phosphorylation-EGFR antibody, Ras antibody, Raf antibody, MEK antibody, phosphorylation-MEK antibody, ERK antibody, phosphorylation-ERK antibody, PI3K antibody, phosphorylation-PI3K antibody, AKT antibody, phosphorylation-AKT antibody or β-actin antibody, 1:1000) overnight at 4 °C, the membranes were washed three times with TBST, and incubated with secondary antibodies (1:5000) at room temperature for 1 h followed by wash with TBST and ECL detection.

### Caspase-3 and caspase-9 activity measurements

KYSE-150 cells were treated with GE11-Ori-Se NPs for 48 h, harvested by scraping, and washed twice with PBS. The cell pellets were then suspended in cell lysis buffer and incubated for 15 min on ice. After centrifugation at 16,000×*g* for 15 min, the collected supernatants were immediately measured to obtain the protein concentration using the Bio-Rad protein assay kit. For caspase activity detection, the cell lysates were added into 96-well plates and then incubated with specific caspase-3 substrates (Ac-DEVD-pNA) and specific caspase-9 substrates (Ac-LEHD-pNA) for 2 h at 37 °C, respectively. Caspase-3 and caspase-9 activities were determined by fluorescence intensity with the excitation wavelength both set at 405 nm using a spectrophotometer.

### KYSE-150 xenograft mice model

Female BALB/c nude mice of 4 ∼ 6 weeks age were obtained from Beijing HFK Bioscience Co (Beijing, China) Ltd and quality checks were supervised by Institute of Laboratory of Animal Science (CAM&PUMC). Harvested KYSE-150 cells suspension (1 × 10^7^ cells in 100 µL PBS) were mixed with 50 µL matrigel and then subcutaneously injected into the right oxters of nude mice. When the average volume of tumors reached about 50–75 mm^3^, mice were randomly divided into 3 treatment groups and a control group (six mice per group). For administration, GE11-Ori-Se NPs treated mice were given with GE11-Ori-Se NPs at dosages of 2.5, 5, and 7.5 mg/kg/d for 15 d through tail intravenous injection and the control group was given with physiological saline by tail intravenous injection. No mice were dead during the whole experimental process. After 15 d administration, the nude mice were sacrificed; the tumors were excised, photographed, and weighted. All experimental protocols were approved by Animal Ethics Committee of Guangdong province (China). And all the experiments were performed in accordance with relevant guidelines and regulations of Animal Ethics Committee of Guangdong province (China). For *in vivo* biodistribution studies of selenium after GE11-Ori-Se NPs administration for 15 d, the mice were sacrificed and tumor, heart, liver, spleen, lung, and kidney were collected and used to determine the selenium content in different tissues using a similar method as reported (Gao et al., 2014). The tissue samples were lyophilized, weighed, and digested with nitric acid and hydrogen peroxide (4:1, v/v) by heating. Then, the sample solution was cooled at room temperature and then analyzed for selenium content using inductively coupled plasma mass spectrometry (ICP-MS, VG TJA, Waltham, MA).

For *in vivo* distribution study of GE11-Se NPs by fluorescence imaging, esophageal cancer bearing mice were injected with coumarin-6-loaded Chi-Se NPs (5 mg/kg), coumarin-6-loaded GE11-Se NPs (5 mg/kg), and coumarin-6-loaded GE11-Se NPs (5 mg/kg) in the presence of large amounts of free GE11 peptide (10 mg/kg) through tail intravenous, respectively. After the mice were anesthetized, whole body fluorescence images were acquired using an *in vivo* image system (Bruker, Karlsruhe, Germany) with excitation wavelength of 488 nm and recorded wavelength at 530 nm.

### Statistical analysis

Statistical analysis was performed using nonparametric t test or ANOVA test, with *p < .05 regarded as statistically significant.

## Results and discussion

### Preparation and characterization of GE11-Ori-Se NPs

**Scheme 1.**
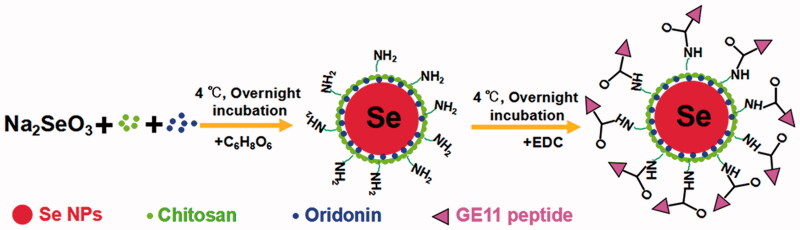
The formation process of GE11-Ori-Se NPs.

Here, we demonstrate a simple method to synthesize GE11-Ori-Se NPs as shown in Scheme 1. Se NPs are loaded with oridonin and further conjugated with GE11 peptide for EGFR targeted oridonin delivery to cancer cells. Chitosan could stabilize selenium element from the reaction between sodium selenite and ascorbic acid to form stable nanoparticles (Chi-Se NPs), which would leave the NH_2_ group at the surface of nanoparticles. Meanwhile, the oridonin molecules added in the colloidal dispersion could be incorporated into Chi-Se NPs to form Ori-Se NPs. At last, the surface NH_2_ groups of nanoparticles could covalently bind to the COOH groups of GE11 peptide to form GE11-Ori-Se NPs.

**Figure 1.**
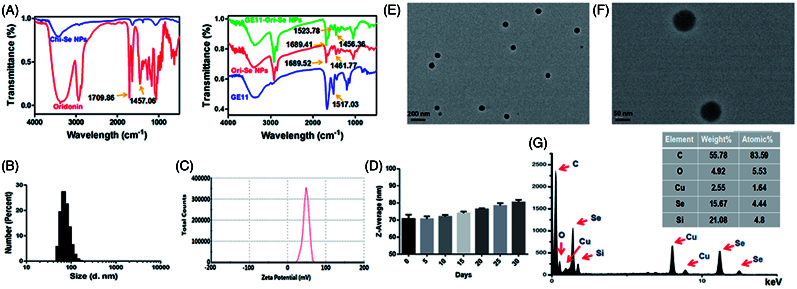
Characterization of GE11-Ori-Se NPs. (A) FTIR spectra of oridonin, Chi-SeNPs, Ori-Se NPs, GE11 peptide and GE11-Ori-Se NPs. (B) Size distribution of GE11-Ori-Se NPs and (C) Zeta potential distribution of GE11-Ori-Se NPs. (D) time-course of size distribution of GE11-Ori-Se NPs. (E,F) High-resolution TEM images of GE11-Ori-Se NPs, scale bars are 200 and 50 nm for (E) and (F), respectively. (G) TEM-EDS analysis of GE11-Ori-Se NPs.

Chemical structure of the obtained nanoparticles was confirmed by FTIR to prove the successful formation of GE11-Ori-Se NPs. As shown in Figure 1(A), GE11-Ori-Se NPs showed characteristic peaks at 1689.41 and 1456.36 cm^−1^ from oridonin. The absorbance peak of oridonin at 1709.86 cm^−1^ was due to the stretching vibration of C=O bond of oridonin and the absorbance peak at 1456.36 cm^−1^ could be attributed to the C=H bond in CH_3_ or CH_2_ groups of oridonin. The dramatic shift absorbance peak of C=O bond in oridonin to 1689.41 cm^−1^ after loading onto Se NPs indicated that the encapsulation processes had stronger effects on the stretching vibration of C=O bond, also suggesting that the coupling of oridonin and Se NPs might induce the formation of the O=Se bond between the ligands and the Se NPs surface. GE11-Ori-Se NPs also showed specific absorption at 1523.78 cm^−1^, which was similar with the specific absorption of GE11 peptide at 1517.03 cm^−1^, confirming the successful conjugation of GE11 peptide onto Se NPs. And the UV–vis spectra analysis also demonstrated the characteristic peaks of GE11 peptide on GE11-Ori-Se NPs (Figure S1). These results indicate the successful loading of oridonin onto Se NPs and the successful decoration of GE11 peptide onto Ori-Se NPs surface.

The size distribution and zeta potential of the nanoparticles were determined by dynamic light scattering. GE11-Ori-Se NPs showed a narrow size distribution from 38 to 135 nm with an average diameter of 70 nm (Figure 1(C)). Besides, GE11-Ori-Se NPs showed an average zeta potential of 48 mV, which promoted the nanoparticle system more easily to be internalized by cancer cells due to the positive charge of Se NPs surface (Yu et al., 2012a). The size distribution and zeta potential of blank Se NPs (Chi-Se NPs) and Ori-Se NPs were also analyzed, which demonstrated that oridonin loading and GE11 peptide surface decoration increased the average particle size of Se NPs from 55 to 60 nm, and finally to 70 nm for GE11-Ori-Se NPs (Figure S2). However, oridonin loading and GE11 peptide surface decoration showed no significant influence on the zeta potential (Figure S3). The average diameter of GE11-Ori-Se NPs in aqueous solution was also investigated in a long duration, which showed no dramatic aggregation of the nanoparticles, demonstrating that GE11-Ori-Se NPs were very stable (Figure 1(D)). The size distribution of GE11-Ori-Se NPs was further confirmed by TEM (Figure 1(E,F)), which showed that GE11-Ori-Se NPs were near-spherical shaped and monodispersed particles with average diameter of 70 nm. Further elemental composition analysis was carried out using TEM-EDS, which showed the presence of a strong signal from the Se atoms (Figure 1(G)). The encapsulation efficacy and loading content of oridonin on GE11-Ori-Se NPs were also determined, which showed an encapsulation efficacy of 5.48 ± 0.27%, and 1 mg GE11-Ori-Se NPs contained 0.15 ± 0.01 μg oridonin (Table S1).

### *In vitro* drug release of GE11-Ori-Se NPs

**Figure 2.**
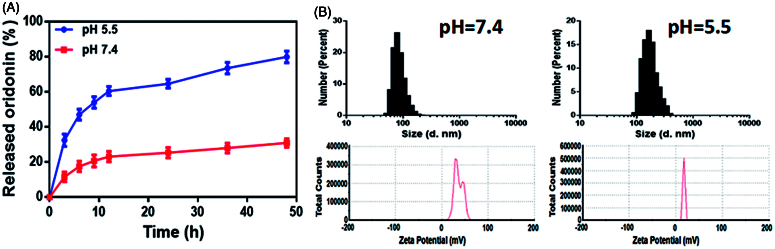
(A) *In vitro* release of oridonin from GE11-Ori-Se NPs at pH 5.5 and 7.4. (B) Size and zeta potential distribution of GE11-Ori-Se NPs in PBS solution (pH 5.5 and 7.4).

The drug release behavior from GE11-Ori-Se NPs was investigated in PBS solution at pH 7.4 and 5.3 to simulate the blood and lysosome environments *in vivo*. As indicated in Figure 2(A), the cumulative release amount of oridonin from the nanoparticles at pH 5.3 was nearly 32.48 ± 2.02% within 3 h and nearly 82.29 ± 0.94% for 48 h, whereas the release rate at pH 7.4 was 11.6 ± 1.57% in 3 h and finally reached 36.95 ± 0.61% for 48 h. These results demonstrated the pH sensitive release of oridonin in GE11-Ori-Se NPs. To further investigate the pH-sensibility of GE11-Ori-Se NPs, DLS was used to explore the variation in the particle size and zeta potential under different external pH (Figure 2(B)). At pH 7.4, the nanoparticles were endowed with a similar hydrodynamic diameter (80 nm) compared to the initial solution (70 nm), and the zeta potential of GE11-Ori-Se NPs slightly decreased to 37 mV. However, in case of pH 5.5, the particle underwent a significant average particle size increase to 190 nm, and the zeta potential of GE11-Ori-Se NPs dramatically decreased to 17 mV. This might be caused by the wrapping of chitosan around the outside of GE11-Ori-Se NPs, which exposed a lot amino groups that were responsive to pH at the surface of nanoparticles. Under neutral and slightly basic condition (pH 7.4), chitosan was orderly aggregated on the surface of Se NPs due to the deprotonation of its amino groups, which hindered the release of oridonin from nanoparticles. However, under acidic condition (pH 5.5), the amino groups on chitosan were protonated, which provided a strong electrostatic repulsion and less effective hydrogen bonding between chitosan molecules (Liu et al., 2015b). In this case, the conformation of chitosan changed, which could destroy the stabilization effects of chitosan on Se NPs, resulting in the dramatically increased particle size, significant decrease of surface zeta potential and rapid leaking out of oridonin. These features of the chitosan layer in GE11-Ori-Se NPs system thus contribute to the ability of controlled oridonin release from GE11-Ori-Se NPs under acidity condition.

### Selective cellular uptake and uptake mechanism of GE11-Se NPs

**Figure 3.**
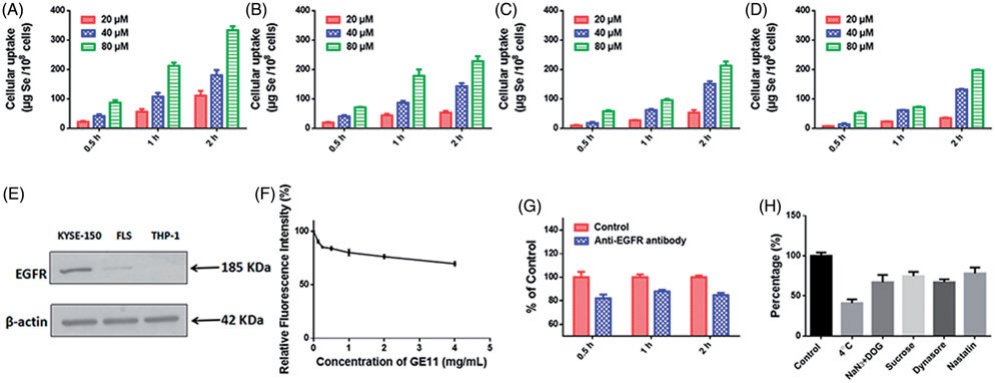
Cellular uptake of GE11-Se NPs. Cellular uptake of (A) coumarin-6-loaded GE11-Se NPs and (B) coumarin-6-loaded Chi-Se NPs in KYSE-150 cells, *n* = 3. Cellular uptake of coumarin-6-loaded GE11-Se NPs in (C) FLS and (D) THP-1 cells, *n* = 3. (E) EGFR expression in KYSE-150 cells, fibroblast-like synovial cells and THP-1 cells. (F) Intracellular uptake of coumarin-6-loaded GE11-Se NPs in KYSE-150 cells with free GE11 peptide competition, *n* = 3. (G) Cellular uptake of coumarin-6-loaded GE11-Se NPs in KYSE-150 cells with anti-EGFR antibody block, *n* = 3. (H) Cellular uptake of coumarin-6-loaded GE11-Se NPs in KYSE-150 cells under different endocytosis inhibition conditions, *n* = 3.

Selective cancer cell targeted cellular uptake of therapeutic drugs is still one of the most attracting methods for cancer treatment. Surface modification of nanomaterials with specific ligands to achieve specific ligand-mediated targeting may be a feasible strategy to enhance the selectivity of drugs against cancer cells. By introducing GE11 peptide onto the surface of Se NPs, the obtained nanoparticles might be an efficient candidate for EGFR targeted cancer treatment. Couramin-6 loaded Chi-Se NPs and GE11-Se NPs, which showed similar diameter size and zeta potential with the Chi-Se NPs and GE11-Se NPs loaded with oridonin (Figure S4), were used to confirm the role of GE11 peptide surface conjugation in cellular uptake of Se NPs. Obtained results demonstrated that the uptake of GE11-Se NPs in KYSE-150 cells (Figure 3(A)) was higher than that of Chi-Se NPs (Figure 3(B)). To examine the selectivity of GE11-Se NPs between cancer and normal cells, we compared the internalization of couramin-6 loaded GE11-Se NPs in EGFR high expressed KYSE-150 cells, EGFR middling expressed human FLS and EGFR low expressed THP-1 cells (Figure 3(E)). The uptake of coumarin-6-loaded GE11-Se NPs in KTSE-150 cells (Figure 3(A)) was much higher than that of FLS cells (Figure 3(C)) and THP-1 cells (Figure 3(D)) at the same treatment conditions, demonstrating the selective uptake of coumarin-6-loaded GE11-Se NPs by EGFR high expressed cancer cells.

To further test the contribution of EGFR in the uptake of coumarin-6-loaded GE11-Se NPs, we then performed GE11 peptide competing assay in KYSE-150 cells. The cells were pretreated with excess amount of GE11 peptide and then incubated with coumarin-6-loaded GE11-Se NPs for various periods of time. As shown in Figure 3(F), free GE11 peptide could significantly inhibit the cellular uptake of GE11-Se NPs, and 4 mg/mL GE11 peptide treatment resulted in nearly 30% reduced uptake of coumarin-6-loaded GE11-Se NPs. Anti-EGFR antibody was also used to block EGFR in KYSE-150 cells, which also resulted in nearly 20% reduced uptake of coumarin-6-loaded GE11-Se NPs (Figure 3(G)). These results strongly suggested that the selective cellular uptake of GE11-Se NPs by cancer cells could be partially traced to EGFR dependent endocytosis in cancer cells.

To further understand the uptake mechanism of coumarin-6-loaded GE11-Se NPs by cancer cells, KYSE-150 cells were pretreated with different endocytosis inhibitors before the addition of coumarin-6-loaded GE11-Se NPs. As shown in Figure 3(H), NaN_3_ in combination with DOG treatment, or low temperature (4 °C), strongly inhibited the uptake of coumarin-6-loaded GE11-Se NPs to 67.04 ± 5.34 and 41.03 ± 2.74% of control, which suggested that coumarin-6-loaded GE11-Se NPs was transported into the cells by means of energy-dependent endocytosis. For non-phagocytic cells, caveolae/lipid raft-mediated, and clathrin-mediated endocytosis are two main mechanism of endocytosis (Wang et al., 2011). Sucrose, a specific inhibitor of clathrin-mediated endocytosis, was found to inhibit the uptake of coumarin-6-loaded GE11-Se NPs to 74.71 ± 3.12% of control, indicating that the clathrin-mediated endocytosis was also involved in the endocytosis pathway of coumarin-6-loaded GE11-Se NPs. Nystatin, an inhibitor of lipid raft-dependent endocytosis, caused a reduction of uptake to 67.25 ± 2.08% of control, demonstrating that lipid raft-mediated endocytosis was also involved in the endocytosis of coumarin-6-loaded GE11-Se NPs. Dynasore, a specific inhibitor essential for dynamin mediated lipid raft endocytosis, induced a reduction of uptake to 78.13 ± 4.23% of control, suggesting that dynamin-mediated pathway was the main pattern of lipid raft-dependent endocytosis of coumarin-6-loaded GE11-Se NPs in KYSE-150 cells. Thus, both lipid raft-mediated and clathrin-mediated endocytic pathway were involved in the cellular uptake of coumarin-6-loaded GE11-Se NPs in KYSE-150 cells.

### Intracellular localization of GE11-Se NPs

**Figure 4.**
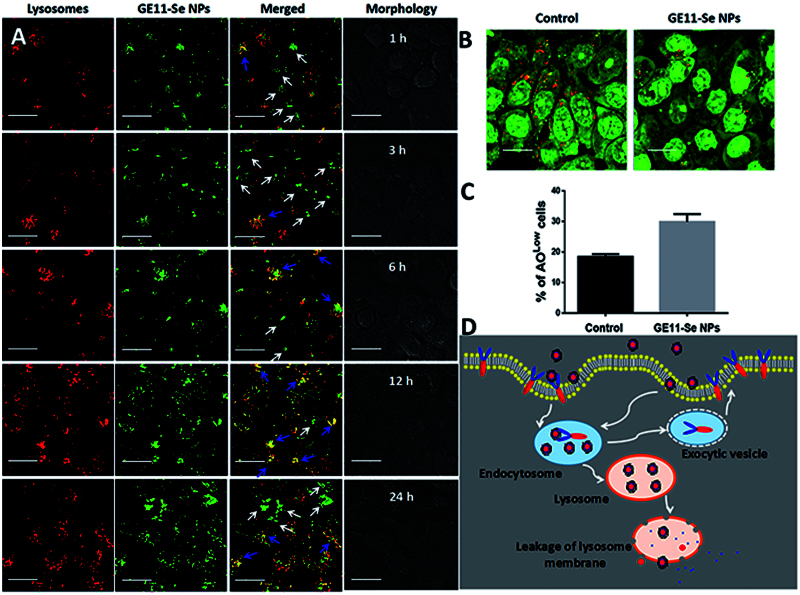
Intracellular localization of GE11-Se NPs. (A) Intracellular trafficking of coumarin-6-loaded GE11-Se NPs. KYSE-150 cells treated with coumarin-6-loaded GE11-Se-NPs (80 μM) were stained with lysotracker (lysosome) or different periods of time and visualized under a confocal microscopy, scale bar: 20 μm. The changes in integrity of the lysosomal membrane (AO staining) in KYSE-150 cells after 24 h GE11-Se-NPs (80 μM) by (B) confocal microscopy and (C) flow cytometry, scale bar; 30 μm. (D) Proposed endocytosis pathway of GE11-Se-NPs in KYSE-150 cells.

The intracellular localization of nanomaterials plays very important roles in the cytotoxicity of nanomaterials because the direct exposure of nanomaterials to some organelles could destroy the structure and function of these organelles (Wang et al., 2011). As the most widely known organelles, lysosomes are found to be closely related to the intracellular localization of nanomaterials, and further act critical roles in the intracellular drug release and toxicity of nanomaterials (Zhou et al., 2010; Wang et al., 2011; Huang et al., 2013). To investigate the fate of GE11-Se NPs in cancer cells, fluorescence imaging was employed to gain more insights into the intracellular localization of couramin-6 loaded GE11-Se NPs in KYSE-150 cells. As shown in Figure 4(A), couramin-6 loaded GE11-Se NPs could accumulate into the lysosomes of KYSE-150 cells in 12 h (The overlay of lysosomes and nanoparticles were indicated by blue arrows), however, most of the couramin-6 loaded GE11-Se NPs weren’t entrapped in lysosomes after 24 h incubation as a lot couramin-6 loaded GE11-Se NPs located outside lysosomes (The separate localization of lysosomes and nanoparticles were indicated by white arrows).

The damage of lysosomal membrane could lead to the translocation of nanomaterials inside lysosomes to other areas of cells. Here, we further used acridine orange (AO) as a probe to study the integrity of the lysosomal membrane. The disappeared red fluorescence detected by confocal microscopy and increased low AO signal cells detected by flow cytometry showed significant changes in lysosomal membrane permeation after internalization of GE11-Se NPs into lysosomes of KYSE-150 cells (Figure 4(B,C)), which provide a chance for GE11-Se NPs to be further released into cytoplasm.

### Selective cancer inhibition effects and enhanced anticancer activity of GE11-Ori-Se NPs

**Figure 5.**
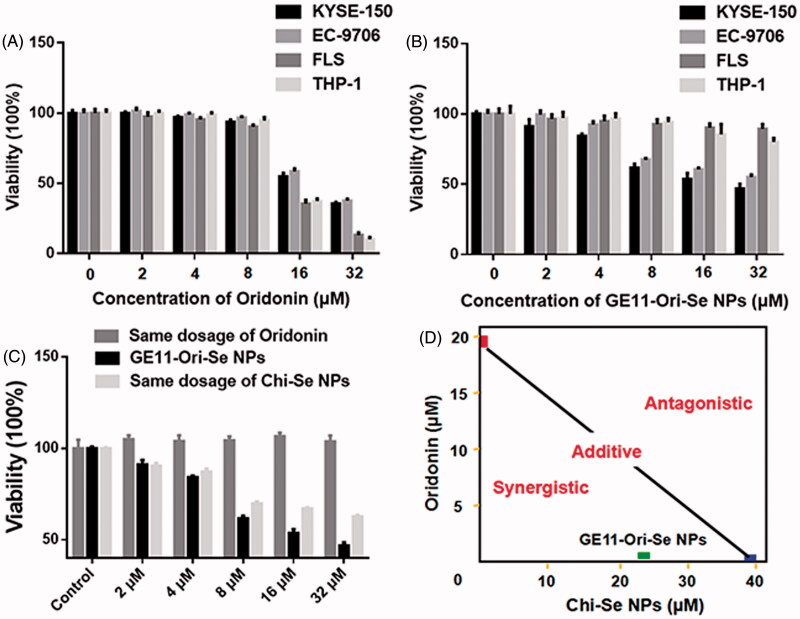
Selective inhibition effects and synergistic/antagonistic analysis of the inhibition effects of GE11-Ori-Se NPs on cancer cells. (A) Effects of oridonin on the viability of human esophageal cancer KYSE-150 cells, human esophageal cancer EC-9706 cells, human fibroblast-like synovial cells and human THP-1 cells, *n* = 3. (B) Effects of GE11-Ori-Se NPs on the viability of human esophageal cancer KYSE-150 cells, human esophageal cancer EC-9706 cells, human fibroblast-like synovial cells and human THP-1 cells, *n* = 3. (C) Effects of GE11-Ori-Se NPs, and the same dosage of oridonin or Chi-Se NPs on the viability of KYSE-150 cells, *n* = 4. (D) Isobologram examination of the growth inhibition effects of GE11-Ori-Se NPs, oridonin and Chi-Se NPs on KYSE-150 cells.

Oridonin has been reported to show very good inhibition effects on cancer cells, but its effects on normal cells are poorly studied. Here, we investigated the effects of oridonin on the viability of human esophageal cancer KYSE-150 cells, EC-9706 cells, human FLS, and human THP-1 macrophages, which demonstrated that oridonin showed higher inhibition effects on the viability of FLS cells and THP-1 cells than that of cancer cells (Figure 5(A)), suggesting the strong toxicity of oridonin against normal cells. As shown in Figure 5(B), GE11-Ori-Se NPs demonstrated dose dependent inhibition effects both on KYSE-150 cells and EC-9706 cells, demonstrating the strong proliferation-inhibition effects of GE11-Ori-Se NPs on cancer cells. However, no significant inhibition effects of GE11-Ori-Se NPs were found in FLS cells and THP-1 cells, suggesting the selective inhibition effects of GE11-Ori-Se NPs on cancer cells. The cytotoxicity of Se NPs (Chi-Se NPs) was also investigated in FLS cells and THP-1 cells, which also demonstrated no significant cytotoxicity of Chi-Se NPs against normal cells (Figure S5).

To clarify the enhanced anticancer activity of GE11-Ori-Se NPs, we also determined the effects of oridonin and Chi-Se NPs corresponding to the same dosage of oridonin and Chi-Se NPs in GE11-Ori-Se NPs on the viability of KYSE-150 cells. As shown in Figure 5(C), the same dosage of oridonin corresponding to the amount of oridonin in GE11-Ori-Se NPs presented no significant effects on the viability of KYSE-150 cells. Chi-Se NPs treatment alone could decrease the viability of KYSE-150 cells significantly, especially in high dosages. However, GE11-Ori-Se NPs showed stronger inhibition effects on the viability of KYSE-150 cells. Isobologram analysis showed that the viability inhibition of combined Chi-Se NPs and oridonin treatment was significantly synergistic, as evidenced by the location of data point in the isobologram far below the line defining as additive effect (Figure 5(D)). Taking all results obtained here into account, the strategy to use a GE11-Se NP as a carrier could be a highly efficient way to enhance the efficacy and selectivity of oridonin.

### Activation of intracellular apoptotic pathways and inhibition of EGFR-mediated signaling pathways by GE11-Ori-Se NPs

**Figure 6.**
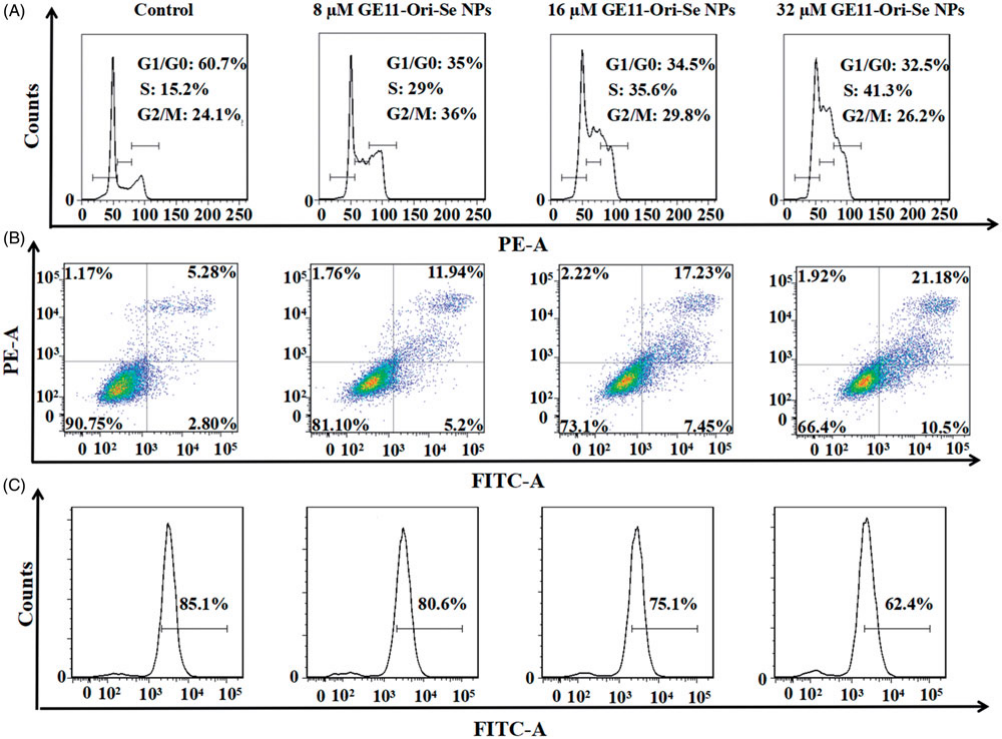
Anticancer effects of GE11-Ori-Se NPs in KYSE-150 cells. (A) Effects of GE11-Ori-Se NPs on the cell cycle of KYSE-150 cells. (B) Effects of GE11-Ori-Se NPs on the apoptosis of KYSE-150 cells. (C) Effects of GE11-Ori-Se NPs on the mitochondrial membrane potential of KYSE-150 cells.

Both oridonin and Chi-Se NPs has been found to have the ability to inhibit cancer cells by arresting the cell cycle and induce apoptosis of cancer cells (Yu et al., 2012b; Estevez et al., 2014; Lu et al., 2017). Thus, we further examined whether cell cycle arrest and apoptosis were also involved in GE11-Ori-Se NPs inhibited cancer cells. The representative cell cycle distribution images demonstrated that the percentage of KYSE-150 cells in G1/G0 phase decreased and the percentage of KYSE-150 cells in S phase significantly increased after GE11-Ori-Se NPs treatment (Figure 6(A)), which implied that cell cycle arrest at S phase might be one of the mechanisms for the anticancer effects of GE11-Ori-Se NPs.

As shown in Figure 6(B), after treated with different concentration of GE11-Ori-Se NPs, the apoptotic KYSE-150 cells increased dose dependently, which demonstrated that cell death induced by GE11-Ori-Se NPs was mainly caused by apoptosis. Cell apoptosis is always associated with the morphological damage of cells. By fluorescence microscopy imaging, we found that the F-actin filaments disappeared with broken or shrunk nuclei in GE11-Ori-Se NPs treated apoptotic KYSE-150 cells (Figure S6). And using atomic force microscopy (AFM), GE11-Ori-Se NPs treated KYSE-150 cells were found to be shrunk with some holes emerged on cell membrane (Figure S7). And the average particle size and roughness for the membrane of KYSE-150 cells both increased after GE11-Ori-Se NPs treatment with dose-dependent manner (Figure S7).

Mitochondria, the functional energy producing organelle of cells, play very important roles in initiation and execution of apoptosis, which is regarded as one of the typical apoptosis pathways (Shi, 2001). To examine the roles of mitochondria in GE11-Ori-Se NPs induced cancer cell apoptosis, we further tested the mitochondrial membrane potential in response to GE11-Ori-Se NPs exposure by flow cytometry. As shown in Figure 6(C), the mitochondrial membrane potential of KYSE-150 cells decreased dose dependently after GE11-Ori-Se NPs treatment, indicating that GE11-Ori-Se NPs could also disrupt the function of mitochondria, which therefore prompting the apoptosis of cancer cells.

**Figure 7.**
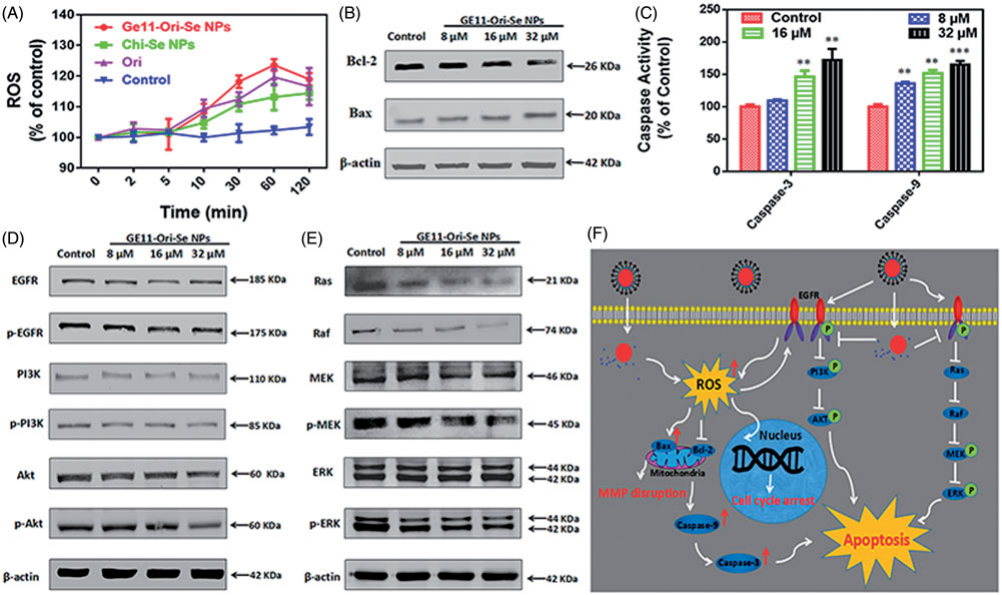
Activation of mitochondria-dependent apoptosis pathway and inhibition of EGFR-mediated PI3K/AKT and Ras/Raf/MEK/ERK pathways by GE11-Ori-Se NPs. (A) Effects of GE11-Ori-Se NPs, and the same dosage of oridonin or Chi-Se NPs on the production of ROS in KYSE-150 cells, *n* = 3. (B) Western blot analysis for the expression of Bcl-2 and Bax in KYSE-150 cells after GE11-Ori-Se NPs treatment. β-Actin was used as loading control. (C) Effects of GE11-Ori-Se NPs on the caspase-3 and caspase-9 activity in KYSE-150 cells, *n* = 3,**p < .01, ***p < .001. (D) Western bolt analysis for the expression of EGFR, p-EGFR, PI3K, p-PI3K, AKT, and p-AKT after GE11-Ori-Se NPs treatment. (E) Western bolt analysis for the expression of Ras, Raf, MEK, p-MEK, ERK, and p-ERK after GE11-Ori-Se NPs treatment. β-Actin was used as loading control. (F) The main signaling pathway of GE11-Ori-Se NPs-induced apoptosis.

As oridonin is a kind anticancer compound that could induce cancer cell apoptosis through ROS-dependent way, we also determined the ROS level upon GE11-Ori-Se NPs treatment. As shown in Figure 7(A), GE11-Ori-Se NPs, Chi-Se NPs and oridonin could all elevate ROS generation in KYSE-150 cells after 5 min treatment, followed by a gradually fall down after 60 min treatment. It was worth to note that GE11-Ori-Se NPs induced higher ROS level than that of oridonin or Chi-Se NPs, suggesting that the combination of Chi-Se NPs and oridonin synergistically further elevated intracellular ROS level in KYSE-150 cells, which therefore resulted in enhanced anticancer activity.

Mitochondrial respiratory chain has been proved to be the main source of intracellular ROS production, which indicates that abnormal ROS production might be attributed to the dysfunction of mitochondria. The Bcl-2 family governs mitochondrial outer membrane permeabilization by Bcl-2 proteins and its homologs, which could be divided into pro-apoptotic or anti-apoptotic. Among the proteins in Bcl-2 family, Bcl-2 is specifically considered an important anti-apoptotic protein and Bax is widely known as a critical pro-apoptotic protein that both involved in mitochondria-dependent apoptosis. As indicated in Figure 7(B), we found that GE11-Ori-Se NPs treatment could decrease Bcl-2 expression and also increase Bax expression in KYSE-150 cells, which indicated that mitochondria dysfunction was also involved in GE11-Ori-Se NPs inhibited KYSE-150 cells.

Caspases, a family of cysteine proteases, are central initiators and executioners of the apoptotic process. Caspase-9 has been identified as the indicator of mitochondria-dependent apoptosis pathways and caspase-3 has been regarded as the downstream effector caspase of apoptosis (Adams, 2003). Here, we also determined the activity of caspase-9 and caspase-3 to further understand GE11-Ori-Se NPs induced KYSE-150 cell apoptosis. As shown in Figure 7(C), both the expression of mitochondria-dependent caspase-9 and apoptosis effector caspase-3 were increased by GE11-Ori-Se NPs treatment in a dose dependent manner. The activity of caspase-9 significantly increased to 164.87 ± 3.30% after treated with 32 μM GE11-Ori-Se NPs for 48 h, and the activity of caspase-3 increased to 171.90 ± 9.97% with 32 μM GE11-Ori-Se NPs treatment. These results collectively suggested that GE11-Ori-Se NPs could induce KYSE-150 cell apoptosis in mitochondria-dependent way.

The overexpression and phosphorylation of EGFR, common mechanisms in tumors of epithelial origin, is associated with poor prognosis, metastasis, and resistance to chemotherapy (Nicholson et al., 2001), which makes it an ideal target for cancer treatment. Here, we further tested the expression and phosphorylation of EGFR, which showed that GE11-Ori-Se NPs inhibited the phosphorylation of EGFR (Figure 7(D)). The activation of EGFR-downstream PI3K/AKT signaling pathway is strongly linked with the protection of cells from apoptosis (Nicholson & Anderson, 2002). By Western blot analysis, we also found that GE11-Ori-Se NPs inhibited the phosphorylation of PI3K and AKT, which demonstrated that GE11-Ori-Se NPs could inhibit EGFR-mediated PI3K/AKT pathway. Despite PI3K/AKT pathway, the classic EGFR downstream Ras/Raf/MEK/ERK pathway is also a key signal transduction pathway of cell proliferation (Roovers & Assoian, 2000). As shown in Figure 7(E), the Ras protein level and Raf protein was significantly reduced after GE11-Ori-Se NPs exposure. Moreover, the results also showed that the treatment of KYSE-150 cells with GE11-Ori-Se NPs resulted in down-regulation in the phosphorylation of MEK and ERK in a dose-dependent manner (Figure 7(E)). These results indicated that GE11-Ori-Se NPs also played the promoting-death role in KYSE-150 cells through inhibiting EGFR-mediated Ras-Raf-MEK-ERK pathway.

### *In vivo* targeting effects of GE11-Se NPs against tumor

**Figure 8.**
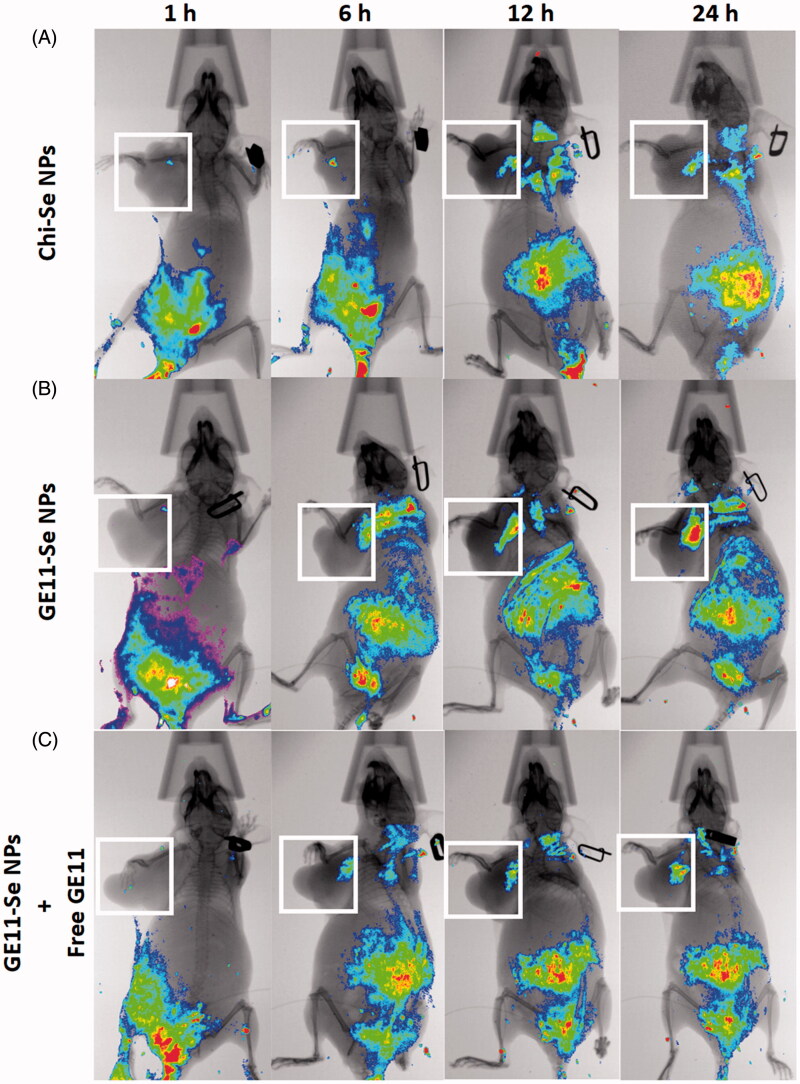
*In vivo* distribution of GE11-Se NPs in xenograft KYSE-150 cancer nude mice. *In vivo* fluorescence imaging of the esophageal cancer-bearing nude mouse after tail intravenous injection of (A) coumarin-6-loaded Chi-Se NPs, (B) coumarin-6-loaded GE11-Se NPs, and (C) coumarin-6-loaded GE11-Se NPs in the presence of large amounts of free GE11 peptide. The white squares indicated the location of tumor.

The targeting ability of GE11-Se NPs in esophageal cancer (KYSE-150 cells) bearing nude mice was estimated using near-infrared fluorescence imaging. Coumarin-6-loaded Chi-Se NPs were used as control to demonstrate the targeting effects of coumarin-6-loaded GE11-Se NPs. As shown in Figure 8(A,B), the fluorescence signal mainly distributed at the abdomen of mice with increased fluorescence signal detected in tumor. However, the fluorescence signal from the tumor was much higher in coumarin-6-loaded GE11-Se NPs treated mice than that of coumarin-6-loaded Chi-Se NPs (Figure 8(A,B)), demonstrating the enhanced targeting effects of Se NPs with GE11 surface decoration. To further discriminate the EPR effects and targeting effects of GE11 peptide on the in vivo targeting effects of GE11-Se NPs, free GE11 peptide were injected into mice together with coumarin-6-loaded GE11-Se NPs, which resulted in decreased fluorescence signal in tumor (Figure 8(C)). These findings suggested that GE11-Se NPs could be served as a potential esophageal cancer targeting agent in tumor-bearing mice due to the EPR effects of Se NPs and the targeting effects of GE11 peptide against tumor.

### *In vivo* anticancer activity of GE11-Ori-Se NPs

**Figure 9.**
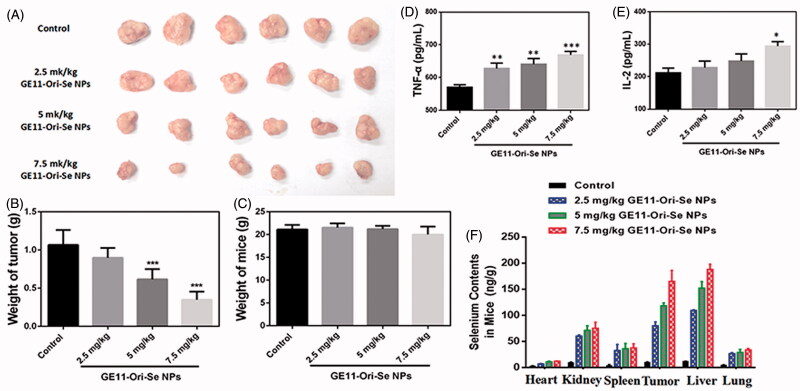
*In vivo* anticancer activity of GE11-Ori-Se NPs in xenograft KYSE-150 cancer nude mice. (A) Images of the tumor from control and GE11-Ori-Se NPs treated xenograft KYSE-150 cancer nude mice. (B) Effects of GE11-Ori-Se NPs on tumor weight, *n* = 6, ***p < .001. (C) Body weight of xenograft KYSE-150 cancer nude mice treated by GE11-Ori-Se NPs, *n* = 6. The data was obtained at the last day of experiment before the sacrifice of mice. Effects of GE11-Ori-Se NPs on the serum (D) TNF-α and (E) IL-2 level in xenograft KYSE-150 cancer nude mice, *n* = 3, *p < .05, **p < .01, ***p < .001. (F) Quantitative analysis of Se contents in different organs in control and GE11-Ori-Se NPs treated mice, *n* = 3.

To observe the anti-tumor activity and systemic toxicity of GE11-Ori-Se NPs, the crucial index for its future medical potential, we applied different concentrations of GE11-Ori-Se NPs in a esophageal cancer (KYSE-150 cells) xenograft nude mice model. At the end of the experiments, the mice were sacrificed and the tumor weight was measured. We found that administration of GE11-Ori-Se NPs for 15 d substantially suppressed tumor growth with few effects on the body weight of mice (Figure 9(A)). Both 5 and 7.5 mg/kg/d GE11-Ori-Se NPs could significantly inhibit tumor growth, and it was also worth to note that nearly 60% of the tumor weight was suppressed by 7.5 mg/kg/d GE11-Ori-Se NPs (Figure 9(B)). Additionally, no significant lose was observed in the body weight of nude mice, indicating the mineral side effect of GE11-Ori-Se NPs (Figure 9(C)). These results demonstrated the effective in vivo tumor-suppressed capacity of GE11-Ori-Se NPs, showing the potential use of GE11-Ori-Se NPs for cancer treatment.

To demonstrate the role of GE11-Ori-Se NPs on the immunity system of tumor-bearing mice, we also tested the serum TNF-α and IL-2 level. TNF-α, a cell signaling cytokine involved in systemic inflammation and tumor development, was found to be up-regulated by GE11-Ori-Se NPs treatment in tumor-bearing mice (Figure 9(D)), which was a common feature of anticancer drug that could enhance the immune system against cancer cells. IL-2, a type of cytokine signaling molecule in the immune system that regulated the activities of white blood cells responsible for immunity, was found to be increased with GE11-Ori-Se NPs treatment (Figure 9(E)). The results suggested that GE11-Ori-Se NPs could also regulate the activity of immune system by changing the cytokine secretion of immunity cells to inhibit tumor growth.

Moreover, we also evaluated the *in vivo* biodistribution of selenium after daily intravenously injection for 15 d by quantitative analyses of the selenium content using ICP-MS to further understand the targeting effects and the metabolism of GE11-Ori-Se NPs. The results (Figure 9(F)) showed the selenium content of all detected tissues or organs in GE11-Ori-Se NPs obviously increased compared with that of control group. Tumor tissues were found to have much more selenium content, which might be due to the targeting effects of GE11-Ori-Se NPs against cancer cells. Liver, a gland played a major role in metabolism with numerous functions in body, was found to gather most of the selenium contents among the tested organs. Liver has been proved to play very important roles in the metabolism of drugs, which could be attributed to the gathering of Se contents in liver. Se NPs had been found to be a potential candidate as a liver protective agent to avoid the liver damage induced by clinical drugs (Gao et al., 2014; Amin et al., 2016). The strong ability of liver in gathering selenium contents might be closely related to the liver protection effects of Se NPs. Kidney also showed significant increase of selenium contents and was found to gather the most contents of selenium apart from tumor and liver, which might be a result of the elimination of selenium by urination. The contents of selenium in lung, spleen, and heart also increased with GE11-Ori-Se NPs treatment, but were less than liver, tumor, and kidney.

**Figure 10.**
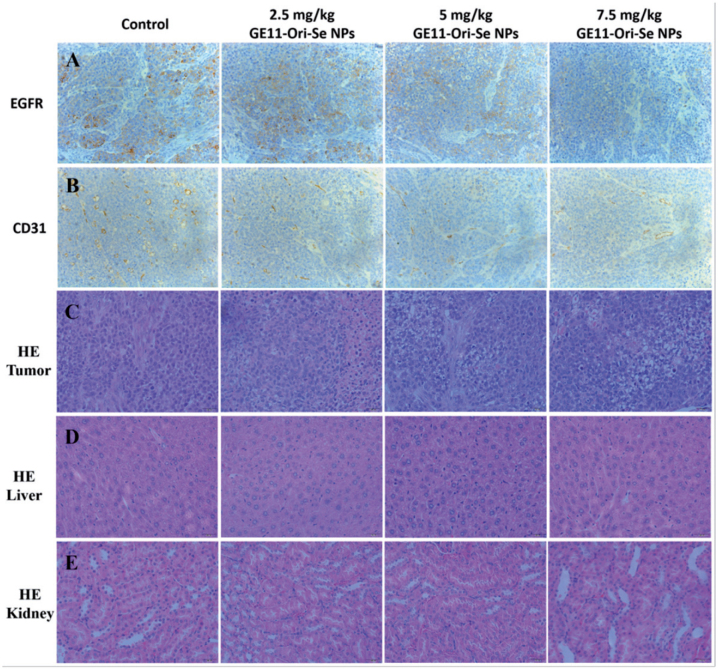
Immunohistochemical analysis of tumor, liver, and kidney from GE11-Ori-Se NPs treated mice. Immunohistochemical analysis of tumor sections from GE11-Ori-Se NPs treated mice by (A) EGFR staining, (B) CD31 staining, and (C) HE staining. HE staining analysis of (D) liver and (E) kidney from GE11-Ori-Se NPs treated mice.

Then, we further tested the expression of some important bio-markers in tumor tissue to investigate the mechanism of tumor inhibition effects of GE11-Ori-Se NPs *in vivo*. The expression of EGFR in tumor tissues is closely related to the malignancy and prognosis of ESCC (Wang et al., 2014; Cui et al., 2015), which urges us to test the expression of EGFR in the tumor tissue upon GE11-Ori-Se NPs. EGFR staining results indicated that GE11-Ori-Se NPs treatment decreased EGFR expression in tumor (Figure 10(A)), implying that GE11-Ori-Se NPs treatment could partially decrease the malignancy potential of ESCC. Tumor angiogenesis is of vital importance to the invasion and metastasis of cancer cells, which therefore has been highlighted as one of the most popular methods for cancer treatment. CD31, a member of the immunoglobulin family involved in angiogenesis, is also found to closely related to the angiogenesis of ESCC (Zhao et al., 2015). Se NPs has been reported to show strong anti-angiogenesis activity (Sun et al., 2013) and oridonin also shows excellent anti-angiogenesis activity (Dong et al., 2014). To determine whether anti-angiogenesis was also involved in the anti-tumor effects of GE11-Ori-Se NPs, we further determined the CD31 expression in the tumor tissue of mice. The obtained results implied that the expression of CD31 in the tumor tissue decreased after GE11-Ori-Se NPs treatment (Figure 10(B)), suggesting that GE11-Ori-Se NPs inhibited tumor growth partially through its anti-angiogenesis effects.

To further confirm the toxicity effects of GE11-Ori-Se NPs *in vivo*, we also applied HE staining to study the structure of tumor, liver and kidney, which were the main organs that gathering selenium contents in mice. The results of HE staining of tumor tissue showed that the tumor after GE11-Ori-Se NPs treatment presented some vacuole-like structures inside, which indicated that GE11-Ori-Se NPs could partially destroy the native structure of tumor (Figure 10(C)). The liver and kidney staining (Figure 10(D, E)) demonstrated that the gathering of selenium had no significant effects on the structure of liver and kidney, suggesting that GE11-Ori-Se NPs were nontoxic to liver and kidney although a lot of selenium contents gathered into these two organs during the metabolism of GE11-Ori-Se NPs.

## Conclusion

In summary, we introduced the synthesis of GE11-Ori-Se NPs as a cancer targeting delivery system to achieve enhanced anticancer efficacy. GE11 surface modification significantly enhanced the cellular uptake of Se NPs through EGFR-mediated endocytosis in EGFR over-expressed cancer cells, which resulted in enhanced inhibition effects against cancer cells and reduced toxicity against normal cells of GE11-Ori-Se NPs. The internalized GE11-Ori-Se NPs could accumulate into lysosomes to release oridonin into cells and then escape from lysosomes after the damage of lysosomal membrane integrity. GE11-Ori-Se NPs were found to induce cancer cell apoptosis by inducting ROS production, activating mitochondria-dependent pathway, inhibiting EGFR-mediated PI3K/AKT and inhibiting Ras/Raf/MEK/ERK pathways. In nude mice xenograft model, GE11-Se NPs showed active targeting effects against tumor. And GE11-Ori-Se NPs was found to significantly inhibit the tumor growth via inhibition of tumor angiogenesis by reducing the angiogenesis-marker CD31 and activation of the immune system by enhancing IL-2 and TNF-α production. This cancer-targeted design of Se NPs provides a new strategy for synergistic treating of cancer with higher efficacy and decreased side effects, suggesting GE11-Ori-Se NPs as a candidate for further evaluation as a chemotherapeutic agent for human cancers, especially for EGFR over-expressed esophageal cancers.

## Supplementary Material

Supplementary_Materials_1_.docx
