# Supplementary material for: GE11 peptide conjugated selenium nanoparticles for EGFR targeted oridonin delivery to achieve enhanced anticancer efficacy by inhibiting EGFR-mediated PI3K/AKT and Ras/Raf/MEK/ERK pathways
Source: Drug Deliv. 2017 Oct 11;24(1):1549–64. doi: 10.1080/10717544.2017.1386729 (PMC6920706; doi:10.1080/10717544.2017.1386729)
Supplement: Supplementary_Materials_1_.docx [file IDRD_A_1386729_SM5988.docx]

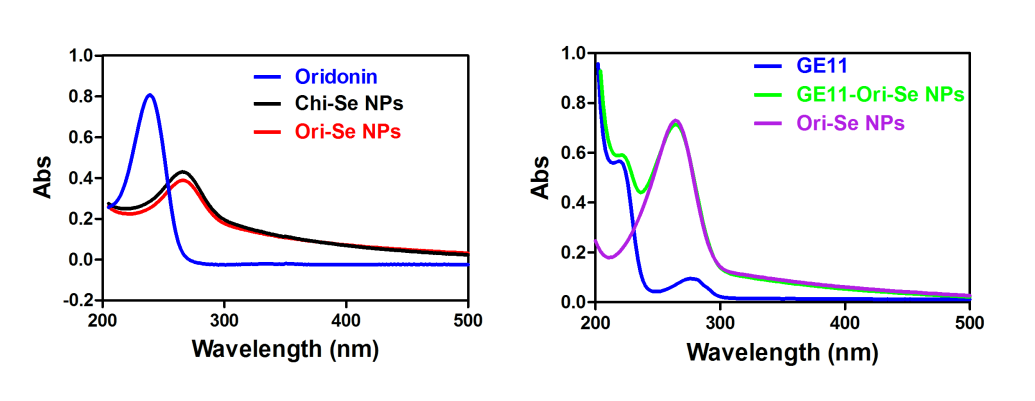


Fig.S1. Ultraviolet absorption spectra of oridonin, Chi-Se NPs, Ori-Se NPs, GE11 peptide and GE11-Ori-Se NPs.


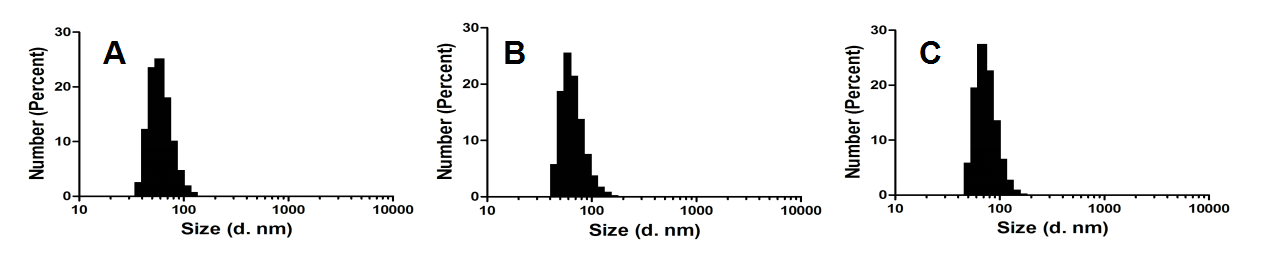


Fig.S2. Size distribution of (A) Chi-Se NPs, (B) Ori-Se NPs and (C) GE11-Ori-Se NPs. Chi-Se NPs, Ori-Se NPs and GE11-Ori-Se NPs showed an average diameter about 55 nm, 60 nm, and 70 nm, respectively.


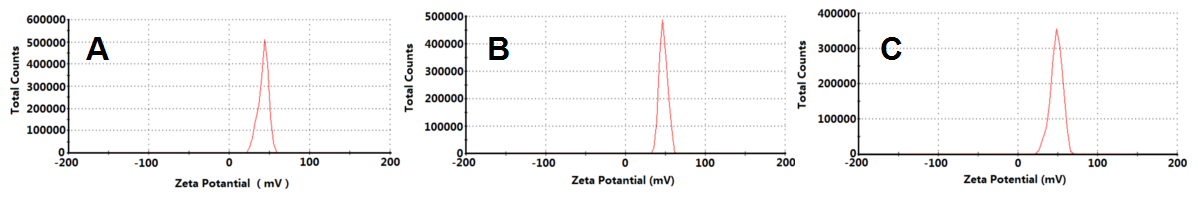


Fig.S3. Zeta potential distribution of (A) Chi-Se NPs, (B) Ori-Se NPs and (C) GE11-Ori-Se NPs. Chi-Se NPs, Ori-Se NPs and GE11-Ori-Se NPs showed an average zeta potential about 44 mV, 47 mV and 48 mV, respectively.


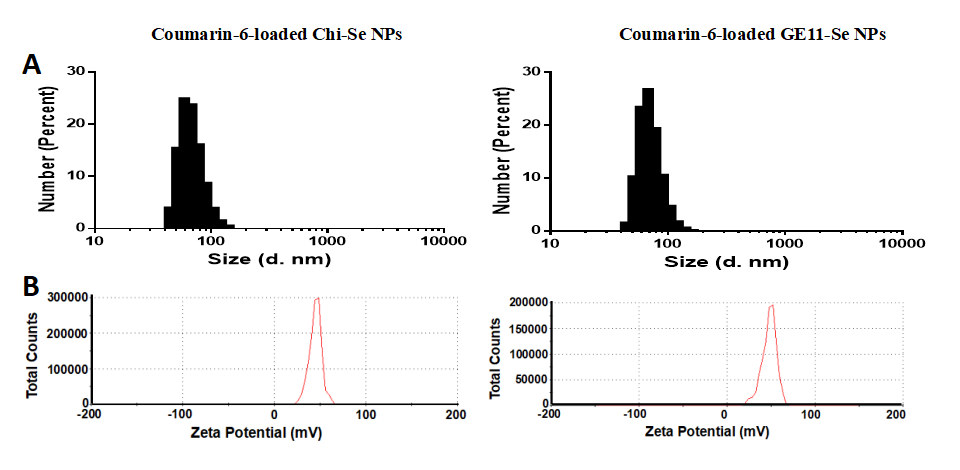


Fig.S4. (A) Size distribution and (B) Zeta potential distribution of coumarin-6-loaded Chi-Se NPs and coumarin-6-loaded GE11-Se NPs. Coumarin-6-loaded Chi-Se NPs and coumarin-6-loaded GE11-Se NPs showed an average size about 60 nm and 70 nm, respectively. Coumarin-6-loaded Chi-Se NPs and coumarin-6-loaded GE11-Se NPs showed an average zeta potential about 45 mV and 48 mV, respectively. Coumarin-6-loaded Chi-Se NPs and coumarin-6-loaded GE11-Se NPs showed similar diameter size and zeta potential with Ori-Se NPs and GE11-Ori-Se NPs.


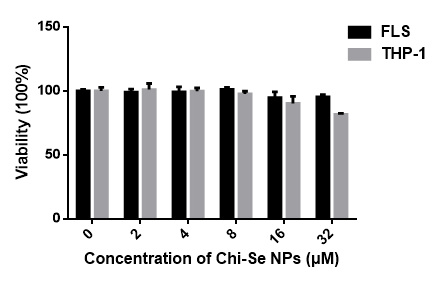


Fig.S5. Effects of Chi-Se NPs on the viability of human fibroblast-like synovial (HFL) cells and THP-1 cells.


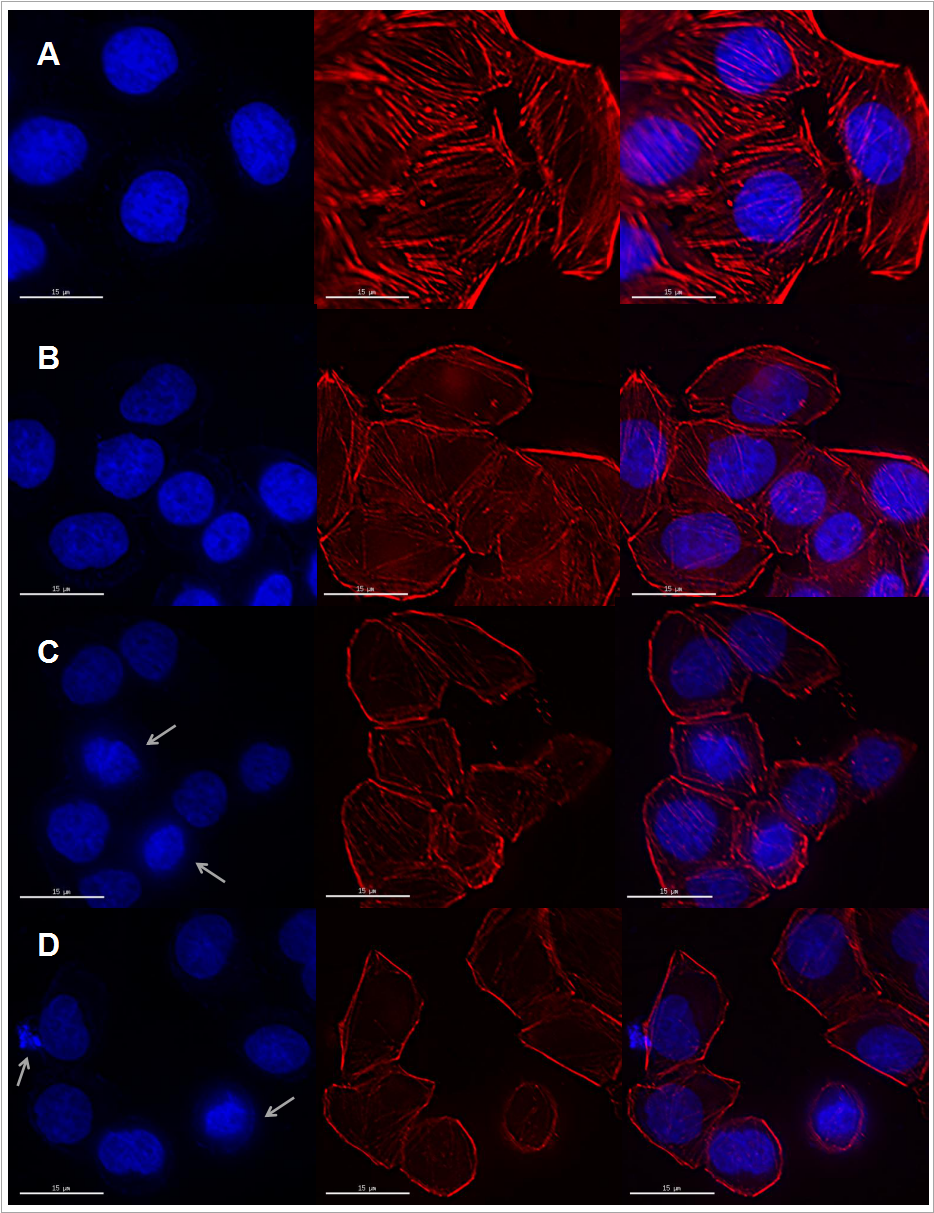


Fig.S6. GE11-Ori-Se NPs induced F-actin disruption in KYSE-150 cells. (A) Control KYSE-150 cells, (B) 8 μM GE11-Ori-Se NPs treated KYSE-150 cells, (C) 16 μM GE11-Ori-Se NPs treated KYSE-150 cells, (D) 32 μM GE11-Ori-Se NPs treated KYSE-150 cells.The white arrows indicated the shrunk or broken nuclei of KYSE-150 cells.


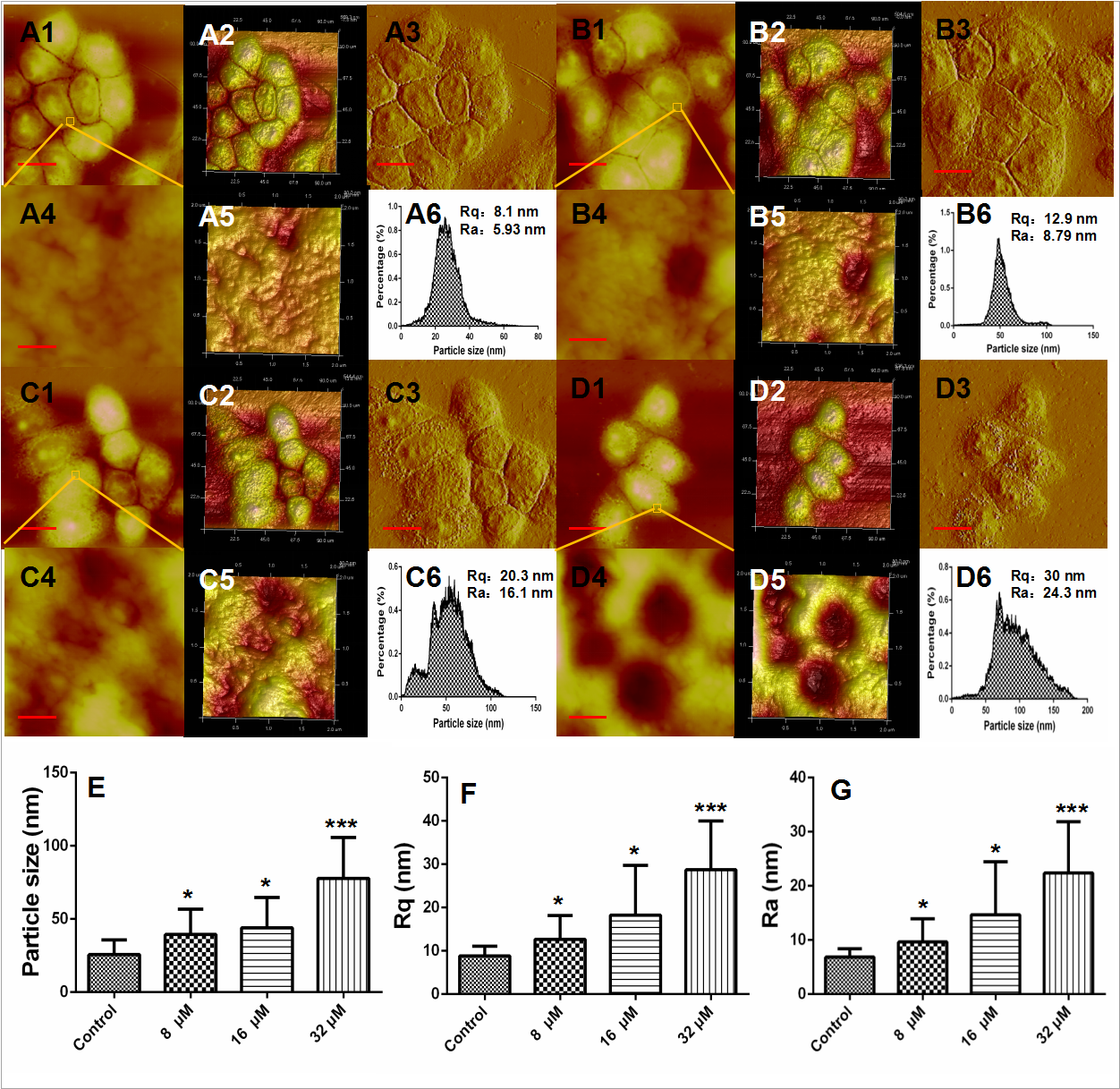


Fig.S7. Effects of GE11-Ori-Se NPs on the morphology and cell surface ultrastructure of KYSE-150 cells. Cell were treated with (A) 0 µM, (B) 8 µM, (C) 16 µM and (D) 32 µM GE11-Ori-Se NPs for 48 h. (A1, B1, C1, D1) Topography of KYSE-150 cells; (A2, B2, C2, D2) 3D images of KYSE-150 cells corresponding to (A1, B1, C1, D1); (A3, B3, C3, D3) Deflection error images of KYSE-150 cells corresponding to (A1, B1, C1, D1); (A4, B4, C4, D4) Topography of KYSE-150 cell membrane obtained from the black frames indicated in (A1, B1, C1, D1); (A5, B5, C5, D5) 3D images of KYSE-150 cell membrane corresponding to (A4, B4, C4, D4); (A6, B6, C6, D6) Particle distribution and roughness analysis of KYSE-150 cell membrane obtained from the images in (A4, B4, C4, D4). Statistical analysis of (E) Particle size; (F) root-mean-squared roughness (Rq) and (G) average roughness (Ra) obtained from KYSE-150 cell membrane, n=12, *p<0.05, ***p<0.001. Scale bars in (A1, A3, B1, B3, C1,C3, D1, D3): 20 µm; Scale bars in (A4, B4, C4, D4): 500 nm.

| **Encapsulation Efficacy** | **Loading Content of Oridonin**  **(1 mg GE11-Ori-Se NPs)** |
| --- | --- |
| 5.48±0.27% | 0.15±0.01 μg |

Tab.S1. Encapsulation rate and loading rate of oridonin in GE11-Ori-Se NPs, n=3.
